# Investigation on Three-Dimensional Void Mesostructures and Geometries in Porous Asphalt Mixture Based on Computed Tomography (CT) Images and Avizo

**DOI:** 10.3390/ma16237426

**Published:** 2023-11-29

**Authors:** Hualong Jing, Hancheng Dan, Hongyu Shan, Xu Liu

**Affiliations:** 1 School of Civil Engineering, Central South University, Changsha 410075, China; jinghualong@csu.edu.cn (H.J.); shanhy@csu.edu.cn (H.S.); liuxu@csu.edu.cn (X.L.); 2 Power China Guiyang Engineering Corporation Limited, Guiyang 550081, China

**Keywords:** porous asphalt mixture, CT technology, Avizo, 3D reconstruction, anisotropy, pore network model

## Abstract

To investigate the void mesostructure in porous asphalt mixtures (PA), computed tomography (CT) and Avizo were utilized to scan and reconstruct the three-dimensional (3D) void model of PA-16 specimens. The void mesostructure of the specimen was quantitatively characterized through the anisotropy evaluation index. The equivalent pore network model (PNM) was extracted using the medial axis method. Based on the PNM model, the topological structure of the specimen and the morphological characteristics of the connected pores were analyzed. The results showed that the void anisotropy evaluation method can reflect the microscopic morphology of voids in porous asphalt mixtures. The cross-sectional porosity of representative elementary volume (REV) is mainly distributed between 20% and 25%, and about 90% of the macropores have a diameter between 0.5 mm and 3 mm. The distribution of cross-sectional porosity is uneven along the REV height direction. As the smallest cross-section of the seepage path, the equivalent radius of the throat is mainly between 0.1 mm and 1.5 mm, which is much smaller than the equivalent radius of the pore. The topological structure of pores is quite different, and their coordination numbers are mainly concentrated within 18. The pores with coordination numbers 1 to 10 constitute the main body of the pores inside REV, accounting for over 98% of the total number of pores. In addition, the permeability calculation results show that there is a significant difference in the permeability of each axis of REV compared to the total permeability of the superpave gyratory compactor (SGC) specimen, which illustrates that the permeability distribution presents an obvious spatial anisotropy. This study effectively reveals the heterogeneity of the 3D void morphology of porous asphalt mixtures, and it provides a reference for a better understanding of the void flow rules in drainage pavements.

## 1. Introduction

Porous asphalt (PA) or open-graded friction course (OGFC) is an environmentally friendly road material due to its noise reduction properties, cleaner rain runoff water, and improved traffic conditions in rainy weather. The performance of a porous asphalt mixture is dependent on its microscopic void (pore) features, which may refer to the content and distribution of its constituents [1]. As we all know, PA is a typical multi-phase composite material that consists of coarse aggregates, asphalt mortar, and voids. It is characterized by an uneven distribution of aggregates and pores, and the void structure has a random geometric distribution in the transverse and longitudinal directions at the mesoscopic scale, making it difficult to analyze the pore space of the mixture in detail. Thus, macroscopic indicators such as average porosity were used to describe its pore characteristics. However, existing research [2,3,4] shows that for the same kind of asphalt mixture, there are significant differences in its mechanical properties and flow characteristics in different directions. It is unreasonable to assume that it is an isotropic material when it produces strain under load. Jiang et al. [5] found that asphalt mixtures with identical air voids may have significantly different performances under given loading and environmental conditions. Zhao et al. [6] showed that asphalt mixtures at the same porosity level exhibit different seepage characteristics, and the spatial distribution and direction of voids have an influence on the flow behavior of the mixture. Liu et al. [7] tested the mechanical characteristics of asphalt mixtures in different directions and found that the anisotropy of the mixture depends on its internal discontinuous structure. Pang et al. [8] explored the difference in hydraulic conductivity of asphalt mixtures in the horizontal and vertical directions through permeability tests. The results indicated that, compared with horizontal hydraulic conductivity, vertical hydraulic conductivity has a more obvious impact on response time and spatial distribution. Therefore, how to obtain void characteristics from the mesoscale and conduct in-depth research on their anisotropy is the key to evaluating the impact of the void structure of a porous asphalt mixture on its macroscopic performance.

Determining the composition and mesostructure of non-transparent objects through advanced image technology has become an effective method for characterizing the microscopic pore structure of porous media [9]. Since the emergence of X-ray CT scanning technology, it has been widely applied not only in medicine but also in many other fields. The microscopic morphological characteristics of the specimen can be non-destructively tested through CT scanning, which is not only convenient and efficient but also has high accuracy in identifying the true void structure of the specimen [10,11,12]. Cui et al. [13] used CT scanning to analyze the internal volume structure of porous asphalt mixtures, which revealed the effect of water damage on the volume change of PA. Li et al. [14] analyzed the impact of porosity distribution on the fatigue performance of the mixture by conducting CT scans on asphalt mixture specimens. Xu et al. [15] studied the influence of void characteristics on the seepage behavior of asphalt mixtures and proved that the void structural characteristics of mixtures are the key to affecting their seepage capabilities. However, the above studies on the void characteristics in asphalt mixtures lack a description of the microscopic morphology of voids and only focus on the size and spatial distribution of voids.

With the rapid development of digital image processing technology, the intersection of multi-disciplinary information in different fields is becoming increasingly close. It is no longer enough to rely on a single field or technology to solve complex problems such as numerical modeling of heterogeneous composite materials and coupled seepage of porous media [16]. Avizo is a powerful visualization software for the fields of earth geological science, materials science, and computer-aided engineering (CAE) engineering calculations. It is mainly used for material characterization and quality control of image analysis and quality assurance, with advanced 3D visualization, image processing, 3D reconstruction, meshing, and other functions. Avizo software has been used by Bird et al. [17] for image segmentation of X-ray tomographic images of rock samples and for studying permeability issues in rock pore structures. Fang et al. [18] utilized computed tomography and Avizo software to construct a three-dimensional meso-equivalent model of reactive powder concrete (RPC), revealing the spatial pore structure characteristics of RPC specimens. Reza et al. [19] employed a digital rock approach to simulate the heat conductivity of micro-CT images of clean sandstone samples by using Avizo software in different directions for evacuated, air-saturated, and water-saturated conditions. The above void characteristics research mostly exists in the field of geotechnical or geological fields, and research in the field of asphalt pavement based on Avizo is rarely involved.

The Pore Network Model (PNM) is an equivalent of the complex pore space structure inside porous media, and it is an abstract model of the pore morphology of real porous media [20]. The model provides an auxiliary approach to studying or predicting the macroscopic properties of porous media by observing and studying the microscopic characteristics of real porous media at the pore scale (such as the position of the pore, the size distribution of pores and throats, etc.) and the topological relationship between pores and throats. Arzilli et al. [21] used digital cores to construct a pore network model of reservoir rocks and obtained rock pore parameters such as throat radius, average throat ratio, coordination number, and shape factor. Li et al. [22] proposed a dynamic pore network model that simulates the 2-phase oil/water displacement during water imbibition in porous media by explicitly modeling the dynamic bulk and film flows within the pores. Existing Avizo and PNM research mostly focuses on soil or rock void structure, and there are few studies on porous asphalt mixtures. How to introduce the above analysis method into the research of asphalt mixtures will be of great significance for accurately revealing the mesoscopic seepage mechanism of drainage asphalt pavement.

In view of the fact that the void structure of a porous asphalt mixture has an important influence on the transmission of fluid within it, the study of the void morphology of the mixture will help to essentially reveal the mecroscopic mechanical behavior and medium transmission characteristics of PA [23]. We will construct a 3D reconstruction model and PNM model of the PA-16 specimen based on CT scan images and Avizo software and conduct a quantitative characterization analysis of the void morphology and topology of the REV. The research results can provide a theoretical basis for further understanding the heterogeneity and void seepage rules of PA pavement.

## 2. Evaluation Method of 3D Void Mesostructures for Porous Pavement

### 2.1. Porous Media Properties of PA Pavement

**Figure 1:**
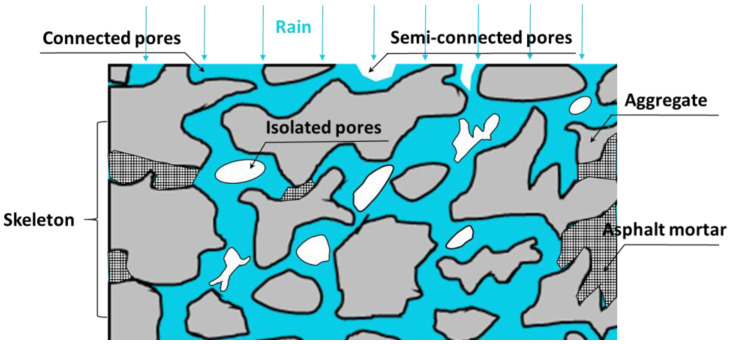
Schematic diagram of the internal structure of drainage asphalt pavement.

Porous media are solid materials that contain a large number of voids, which are widely present in nature, human production, and life, such as soil, rocks, animal and plant organisms, PA pavements, etc. Xiong et al. [24] and Luo et al. [25] made the following framework definition of porous media based on its constituent elements and construction methods:

(a) As shown in Figure 1, porous media is a collection of interconnected units composed of a large number of solid substances (such as mineral particles in porous asphalt mixtures), which are continuous on the macroscopic level and randomly distributed on the microscopic level.

(b) The space in a porous medium is filled with a type of substance or multi-phase substance, where at least one phase of the substance is non-solid and can be a gas or liquid phase. The sum of interconnected solid-phase units in porous media is called the solid-phase matrix (skeleton or porous matrix), and the remaining components are called voids or pores.
(c) As a porous medium, the pore structure of a porous asphalt mixture can be divided into three types according to the type of connection between the pores and the external space, namely connected pores, semi-connected pores, and isolated pores [26]. For porous asphalt mixtures with drainage properties, the disconnected pores can be considered part of the solid matrix. As a matter of fact, isolated pores and semi-connected pores are ineffective at allowing fluid to pass through porous media.

### 2.2. Void Anisotropic Distribution Characteristics

Generally, the differences in mechanical properties of materials in different directions are called anisotropy. According to the different causes of anisotropy, anisotropy is divided into two forms: intrinsic anisotropy and stress-induced anisotropy [27,28], which was first proposed by Casagrande. He believed that intrinsic anisotropy is an inherent property of materials and has nothing to do with additional strains. For asphalt mixtures, the intrinsic anisotropy is formed by the different arrangements of aggregates and voids in each direction during the compaction process, while stress-induced anisotropy refers to physical properties related to strain caused by external loads. For example, cracks may appear inside the mixture after being damaged by stress, which will lead to differences in the mechanical properties of the mixture in different directions.

Although the above two forms of anisotropy exist in asphalt mixtures, when analyzing the void mesostructures of drainage asphalt pavement, if the structural destruction process of the mixture is not involved, only the intrinsic anisotropy of the porous asphalt mixture needs to be considered [29]. In addition, research on the intrinsic anisotropy of asphalt mixtures found that its anisotropy mainly manifests as transverse isotropy, that is, the mechanical properties of asphalt mixtures are mainly different in the vertical and horizontal directions, while there is no significant difference in each direction within the horizontal plane [30,31].

### 2.3. Mesoscopic Evaluation Index of Anisotropy

#### 2.3.1. Porosity

The porosity refers to the ratio of the void volume to the total volume of the sample. By counting the area of voids in a single CT image and the total image area, the porosity of each section is obtained by dividing the void area by the total area of the image. The total porosity of the specimen is the average of the porosity of all images. The specific calculation formula is as follows:

(1) $$\varepsilon =\left[\sum_{i=1}^{n}\frac{{\left(A_{v}\right)}_{i}}{A}\right]n^{-1}$$

where *A_v_* is the void area in a single-slice CT image (mm^2^), *A* is the total area of a single-slice CT image (mm^2^), *ε* is the porosity (%), and *n* is the total number of images.

#### 2.3.2. Connected Porosity

**Figure 2:**
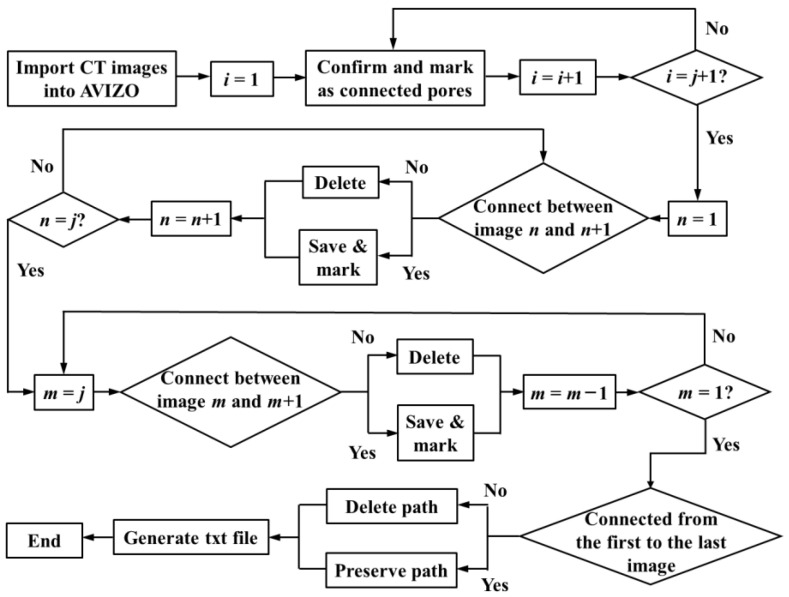
FORTRAN algorithm for analyzing air void connectivity.

Pore connectivity is an important material property of PA mixtures, which is the channel and prerequisite for generating seepage [32]. Due to the randomness and anisotropy of the spatial distribution of the PA mixture pore structure, when analyzing the vertical (*z*-axis) connected pores, it is required to track all connected pores from the top surface to the bottom of the specimen and eliminate all disconnected pores. As shown in Figure 2, connected pores are identified through the FORTRAN algorithm [33,34], where *i*, *j*, *m*, and *n* are all image numbers.

In general, the grayscale value of the asphalt mixture in the CT image ranges from 0 to 255, and the values of each pixel represent the material density of the asphalt mixture at that location. If the pixel points are pores, the corresponding value in the binary value is 0. This principle is used to identify connected pores in each section of the image in the 3D model. When the value is 0, the nearest 8 values around it are checked. If there is a value of 0 among these 8 values, classify all values in the area with a value of 0 as one category and confirm that they are the same. Then, the connectivity of the vertical pores is determined through this algorithm. Starting from the first image on the top, for any pore object, check the values at the same location and the 8 values adjacent to that location in the next image. As long as there is a value of 0 in these areas, it is considered that the pore is connected in these two images, and these two pores are marked as the same pore. Repeat the same steps until the last image is verified. In addition, to calculate the tortuosity of the throat inside the specimen, repeat this step, starting from the last image of the bottom surface to the first image of the top surface. If the pores are not completely connected in these images, they are defined as closed pores, and the closed pores are eliminated from the calculation analysis.

#### 2.3.3. Equivalent Diameter of Pore Throat

The pore diameter can characterize the volume characteristics of the connected pores of the drainage asphalt pavement [35]. The larger the diameter of the connected pores, the less resistance the fluid encounters when flowing in the pores, and the easier it is for fluid flow to occur. Considering that in the calculation process of the diameter of connected pores, the cross-sections (throats) of connected pores are mostly irregular geometric figures, usually connected pores are equivalent to circles with equal areas, and the diameter of the circle is the equivalent diameter (*d*_pore_) of the pore throat. The formula is as follows:

(2) $$d_{\mathrm{pore}}=2\sqrt{S_{\mathrm{pore}}/\pi N}$$

where *S*_pore_ is the total area of connected pores in each cross-sectional image (mm^2^), and *N* is the number of connected pores.

#### 2.3.4. Tortuosity

Due to the complex internal pores of asphalt mixtures, water mostly flows non-linearly within them. Tortuosity is used to characterize the nonlinearity of its seepage path. The formula is as follows:

(3) $$T=\frac{L_{e}}{L}$$

where *L_e_* is the actual length of the water flow (mm) and *L* is the linear vertical length of the water flowing through the outlet and inlet of the specimen (mm).

## 3. CT Scan and Three-Dimensional Reconstruction of Pores

### 3.1. Sample Preparation

**Figure 3:**
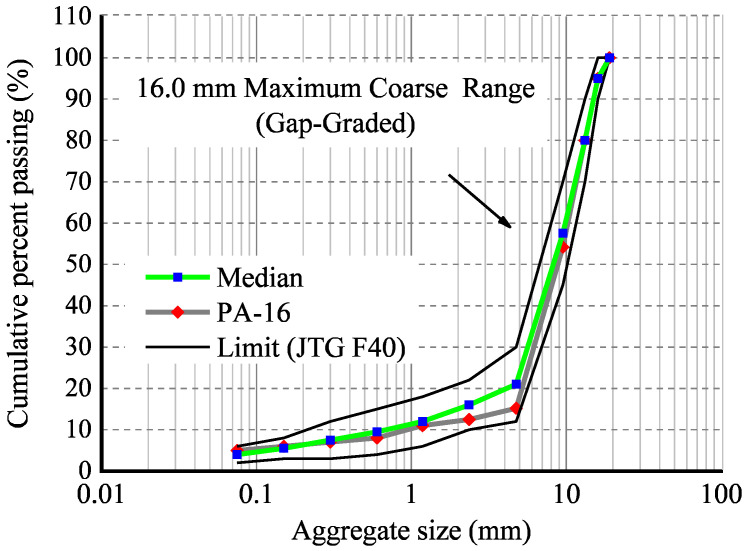
Mineral aggregate size distribution.

The test method refers to China’s Standard Test Methods of Bitumen and Bituminous Mixtures for Highway Engineering (JTG E20-2011) [36] and the Technical Specification for Permeable Asphalt Pavement (CJJ/T 190-2012) [37]. The specimens are prepared by a Superpave gyratory compactor (SGC), and its size (*φ* × *h*) is 100 mm × 100 mm. The coarse aggregate employed in this paper is limestone, and the fine aggregate and filler are granite. The gradation of the PA-16 mixture is designed based on the design steps of an OGFC [36]. The aggregate gradation of the asphalt mixture is illustrated in Figure 3, and the basic physical parameters of the specimen are shown in Table 1.

### 3.2. CT Scanning Test

**Figure 4:**
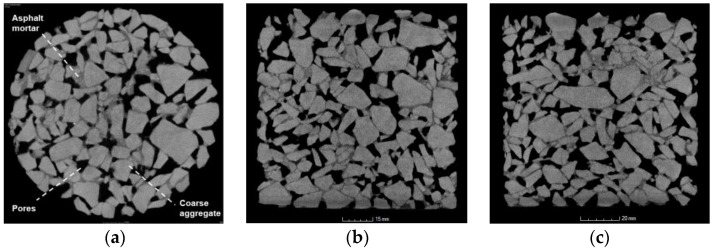
Original CT cross-sectional image: (**a**) plan view; (**b**) side view 1; (**c**) side view 2.

The composition and mesostructure of non-transparent objects can be non-destructively detected using X-ray CT technology. The radiation absorption rate of substances with different densities is defined as the CT number, and the conversion relationship between the density of the detected object and the CT number is established and converted into the corresponding grayscale value. For multi-component composite materials, CT scanning can be used to obtain and evaluate the mesostructures of each component [38]. The CT equipment used in this experiment was the GE Vtomex high-precision industrial CT scanner, the scanning parameters of which are shown in Table 2. The scanning interval is 65 μm, and the CT cross-sectional image obtained by scanning is shown in Figure 4.

### 3.3. Image Processing

As shown in Figure 4, the asphalt mixture is composed of differential substances such as coarse particles, asphalt mortar, and voids. In the original CT images of the collected asphalt mixture specimens, the coarse aggregate is gray, the asphalt mortar is gray-black, and the voids are black. The boundary images are blurred due to the mutual infiltration of various components. In order to solve the problem of target material boundary detection in image processing, this study simplifies coarse aggregate and asphalt mortar into a solid porous matrix and treats the pores as porous media for two-phase processing. The main steps of image processing were as follows.

#### 3.3.1. Image Noise Reduction

**Figure 5:**
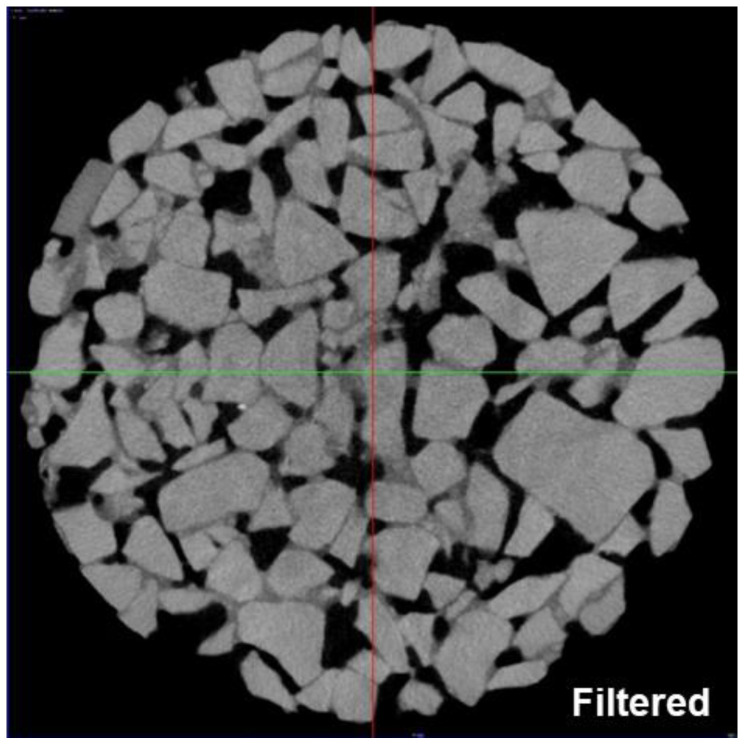
Two-dimensional-image from CT data after image noise reduction. The image corresponds to the same sample location (slice No. 760), which is also shown in Figure 4a before image noise reduction. The sample diameter is 100 mm. Pores = black; aggregates = bright gray; mastic = dark gray.

Since CT images are easily affected by equipment and the external environment during imaging or transmission and often produce image noise, it is necessary to use the Median Filter function and Sobel operator of Avizo software to perform image filtering and image edge detection on the original CT images before image segmentation. The purpose of this operation is to smooth and suppress image noise. The 2D CT cross-sectional image after image noise reduction is shown in Figure 5.

#### 3.3.2. Determine the Optimal Threshold

The Gonzalez iteration method, maximization of interclass variance method (also called Nobuyuki Otsu method, or Otsu method), and average gray value method are commonly used to calculate the grayscale threshold of CT images [39]. The global thresholds obtained by using the above three methods through the Matlab image processing toolbox and Avizo are *T*_1_ = 68, *T*_2_ = 66, and *T*_3_ = 54, respectively. Considering that the more accurate the threshold, the higher the accuracy of the image processing, the better the 3D reconstruction effect of the pores, in order to improve the accuracy of the threshold, as shown in Table 3, based on the principle of minimizing the error between the calculated effective porosity of the 3D reconstruction model and the test value, the interpolation method is used to select the thresholds *T*_4_ = (*T*_2_ + *T*_3_)/2, *T*_5_ = (*T*_1_ + *T*_3_)/2, *T*_6_ = (*T*_2_ + *T*_4_)/2, *T*_7_ = (*T*_2_ + *T*_5_)/2, and *T*_8_ = (*T*_2_ + *T*_7_)/2 for iterative optimization of the optimal threshold.

Table 3 is the effective porosity of the 3D reconstruction model of the REV under *T*_1_~*T*_8_ thresholds based on the image processing function of Avizo software. Compared with the test effective porosity of 21.76%, the calculated effective porosity corresponding to threshold *T*_7_ has the highest accuracy with an error of 0.09%, indicating that the 3D model reconstructed using threshold *T*_7_ has the highest applicability. Therefore, the optimal threshold for image segmentation is 64.

#### 3.3.3. Image Segmentation Using Avizo

**Figure 6:**
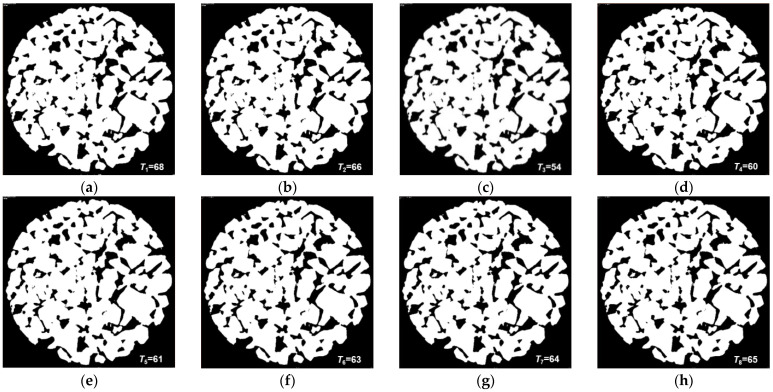
Cross-sectional images segmented with different thresholds: (**a**) image processed using Gonzalez iterative threshold *T*_1_; (**b**) image processed using Otsu method threshold *T*_2_; (**c**) image processed using cross-sectional average gray value method threshold *T*_3_; (**d**–**h**) are images processed using thresholds *T*_4_~*T*_8_, respectively.

After the optimal threshold selection is completed, import all TIFF-format images used for 3D model reconstruction of the specimen into Avizo and perform image segmentation through the Interactive Thresholding function of the software. Taking the 2D cross-sectional image after image noise reduction in Figure 5 as an example, Figure 6 shows the cross-sectional image after *T*_1_~*T*_8_ different threshold segmentation processing.

### 3.4. Three-Dimensional Reconstruction of Pores

**Figure 7:**
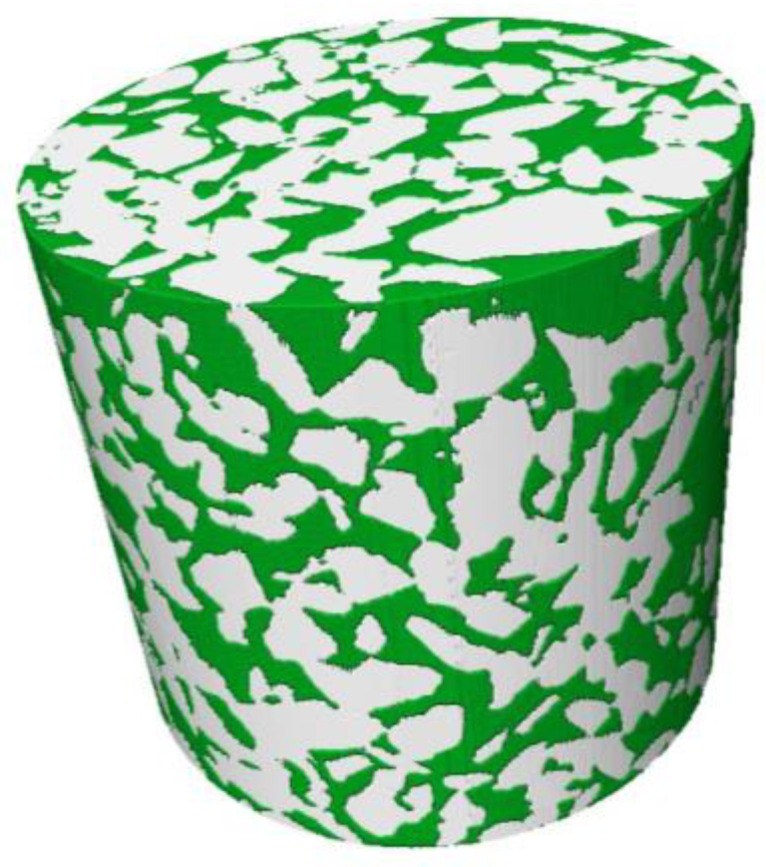
Three-dimensional visual numerical model of the PA-16 specimen.

The three-dimensional visualization numerical model of the PA-16 specimen based on the Volume Rendering function of Avizo is shown in Figure 7. The gray part in the figure represents the solid porous matrix (aggregates and asphalt mortar), while the green part represents the pores.

**Figure 8:**
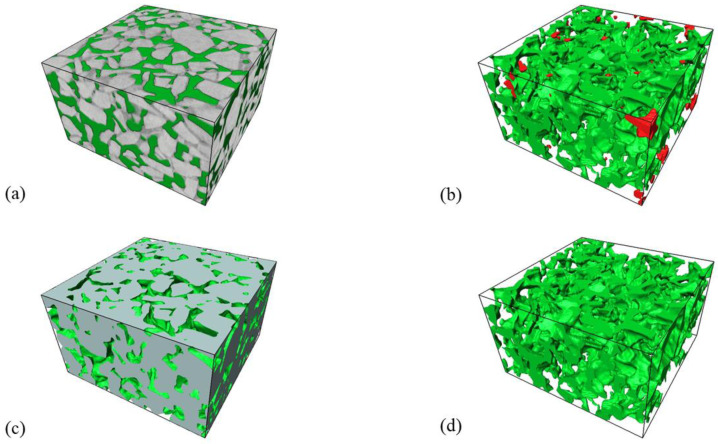
Three-dimensional reconstruction model: (**a**) REV-0 of the specimen; (**b**) REV-1 for all pores; (**c**) REV-2 for solid porous matrix; (**d**) REV-3 for connected pores. Different clusters of pores are shown in different colors. Only the large green pore connects across the sample and was used in characterization.

The real three-dimensional void mesostructure inside the porous asphalt mixture is extremely complex. In order to improve the extraction accuracy of the pore morphology evaluation parameters in the model, this study is based on the mesomechanical analysis method [40] and uses Avizo to intercept the REV in the 3D model of the specimen in Figure 7 as the research object. As shown in Figure 8a, in order to maximize the representation of the structural characteristics of the entire specimen by the captured REV-0, a rectangular parallelepiped was cut out from the middle of the cylindrical specimen using a circle-inscribed square method, with a size of 40 mm × 70 mm × 70 mm (*h* × *l* × *w*). As presented in Figure 8b, the pore model REV-1 is extracted based on the REV-0 model using the Axis Connectivity algorithm. In addition, the solid porous matrix model REV-2 is extracted using the NOT algorithm based on REV-1 (see Figure 8c). The gray part in the figure is the solid porous matrix, and the green part is the connected pores. Figure 8b shows that there are relatively few isolated pores with smaller pore sizes in REV-1 (red part of the figure). Due to the small impact of isolated pores on the overall pore connectivity of the specimen [41], in order to enhance the extraction accuracy of the pore network model, this part of the isolated pores can be cleared through the Remove Islands function of Avizo software. The specific operation process is: Segmentation Editor → Remove Islands → 3D Volume → Highlight All Islands → Apply. The connected pore model REV-3 after deleting the isolated pores is shown in Figure 8d.

## 4. Results and Discussion

### 4.1. Quantitative Characterization of Pore Mesostructures

**Figure 9:**
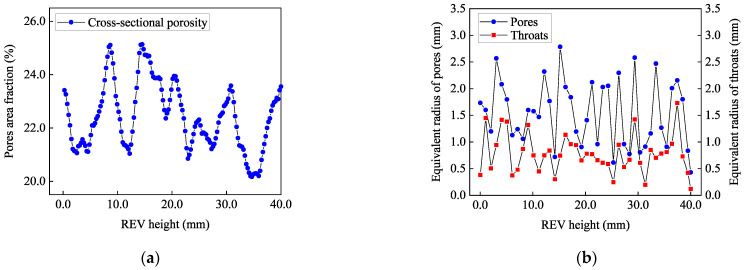
The distribution curves are: (**a**) cross-sectional porosity; (**b**) ER of pores and throats.

As shown in Figure 9, based on the Axis Connectivity, Watershed, and Generate Pore Network Model (GPNM) algorithms, the distribution curve of REVs cross-sectional porosity and the equivalent radius (ER) of the pore and throat were calculated. The specific formula for calculating the cross-sectional porosity *p* is as follows:

(4) $$p=\frac{A_{V}}{A_{T}}$$

where *A*_V_ is the pore area in the single-slice CT image (mm^2^), and *A*_T_ is the total area of the single-slice CT image (mm^2^).

It can be seen from Figure 9a that along the REV height direction, the porosity of each section is mainly distributed between 20% and 25%, and the distribution is uneven along the REV height direction. This result is consistent with the existing literature [42] on the distribution of internal cross-sectional porosity in SGC specimens. In addition, as shown in Figure 9b, the equivalent radius of pores is mainly distributed between 0.5 mm and 3.0 mm, accounting for approximately 89.4% of the total pores ER. The pore throat, as the smallest cross-sectional area in the seepage path, the ER of throats is mainly between 0.1 mm and 1.5 mm, accounting for more than 79.5% of the ER of all throats, and the ER of the throat is about half of the ER of the pore.

**Figure 10:**
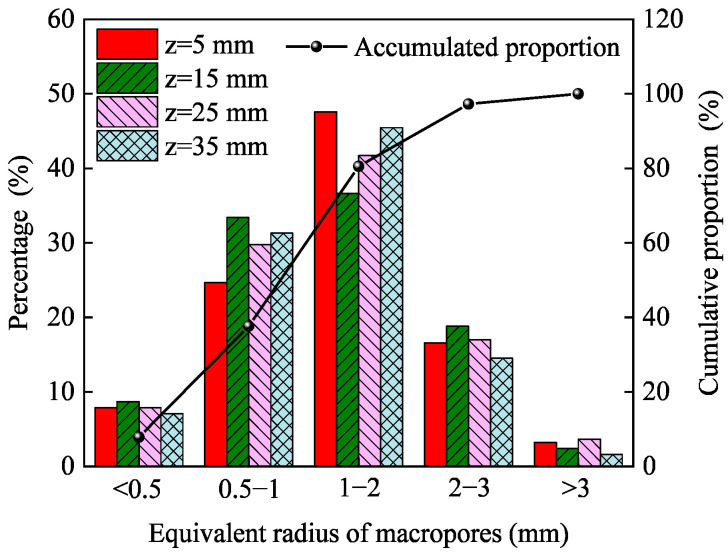
The percentage and cumulative proportion curve of macropores.

In addition, in order to quantitatively characterize the distribution of pores inside the PA mixture, the REV-3 was used as the research object, and according to the ER of macropores, pores were divided into five levels: <0.5 mm (level 1), 0.5 mm~1 mm (level 2), 1 mm~2 mm (level 3), 2 mm~3 mm (level 4), and >3 mm (level 5). Figure 10 shows the statistics of the number of macropores at different levels and the cumulative proportion in REV-3. It can be seen from the figure that the macropores are mainly distributed in levels 2 and 3, and the correlation between the number of macropores and the depth of the REV is not obvious. From a quantitative perspective, the proportion at levels 2, 3, and 4 is higher than 89.0%, and the proportion of macropores in the other two levels (level 1 and level 5) to the total number of pores (1977) is 7.9% and 2.7%, respectively, which indicates that the number of macropores is mainly concentrated between the 2nd and 4th levels, while the ER of macropores in other levels is distributed discretely.

### 4.2. Topological Structure Analysis of Pore Network Model

**Figure 11:**
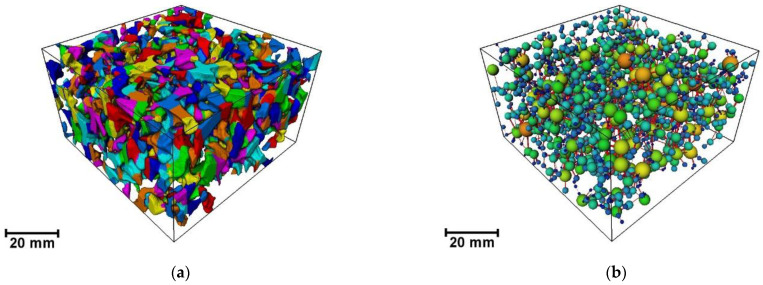
Topological structure: (**a**) morphology of connected pores; (**b**) PNM model. Different colours are automatically presented through the Adjust range option of the PNM View of Avizo.

As shown in Figure 11, based on the connected pore REV-3 reconstruction model, a PNM model (also known as the ball-and-stick model) that reflects the void morphological characteristics of the porous asphalt mixture is extracted using the medial axis method and the Generate PNM algorithm. This model divides the void space into a connected pore-body structure (relatively macropore spaces at the throat connection) and a throat structure connecting the pores [43].

Figure 11 shows the spatial topology of the pores and throats. By rendering different pore sizes in different colors, the connection between the pores and throats can be clearly seen. Furthermore, the topology structure can be quantitatively reflected by the pore coordination number (see Table 4), which represents the number of connections between one pore and surrounding pores, and which is the number of ball sticks around the sphere in Figure 11b.

**Figure 12:**
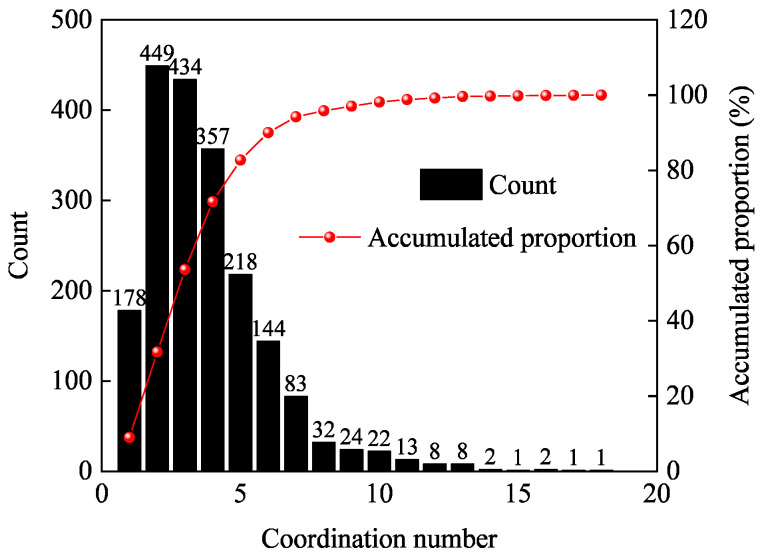
Distribution of the pore coordination number.

In Table 4, we can find that the maximum value of the coordination number in the PNM is 18, which means that the coordination number in the REV-3 is mainly concentrated within 18. Therefore, this paper only counts coordination numbers within 18 when studying the distribution of coordination numbers. As shown in Figure 12, pores with coordination numbers of 1 to 10 constitute the main body of PA mixture pores, accounting for more than 98% of the total pores. The distribution of pore coordination numbers exceeding 10 is in the form of discrete points, and the number is mostly single digits. For drainage asphalt pavement, the greater the coordination number, the more channels for conducting pore flow formed after the pore is connected to the surrounding pores, the stronger the permeability of water inside, and the better the drainage performance of the porous asphalt pavement. Therefore, in order to improve the drainage performance of the practical pavement, in the early stage of porous asphalt mixture mix design, high-viscosity modified asphalt with higher dynamic viscosity can be used to improve the spalling resistance of coarse aggregates and reduce fine aggregates and mineral powder as much as possible. The purpose is to generate more large pore coordination number topological structures within the skeleton void structure of the mixture to form greater connected porosity.

**Figure 13:**
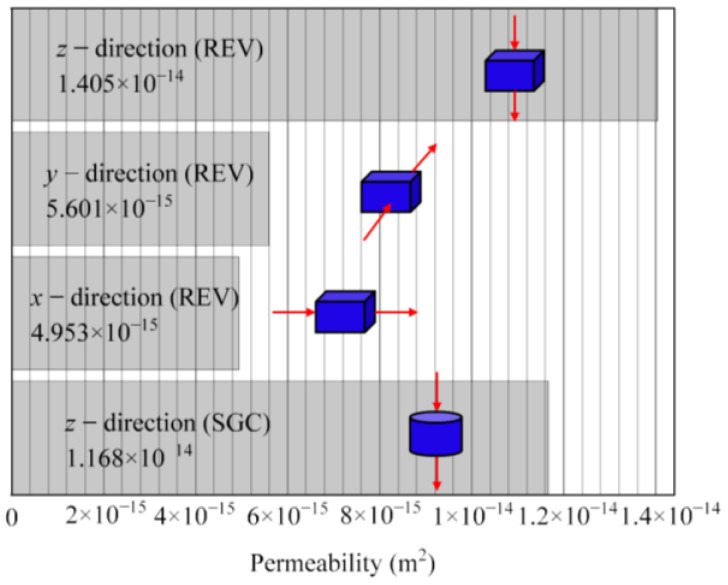
Permeability of REV and SGC specimens. The *x*, *y*, and *z* directions respectively indicated by the red arrows in the figure.

The permeability of REV in the 3D spatial coordinate system is calculated based on the Kozeny–Carman equation (KC equation) of porous media permeability in the field of rock seepage. As shown in Figure 13, the permeability of REV-3 in the *x*, *y*, and *z* directions are 4.953 × 10^−15^ m^2^, 5.601 × 10^−15^ m^2^, and 1.405 × 10^−14^ m^2^, respectively. Compared with the total vertical permeability (1.168 × 10^−14^ m^2^) of SGC specimens, the permeability of the PA mixture exhibits significant spatial anisotropy. By comparing the topological structure of PNM, it can be found that due to the anisotropy of pavement pores, the more connected pores, larger pore throats, and greater permeability, the better the drainage performance of the pavement. Therefore, when paving drainage asphalt pavement, in order to ensure that the pavement has good drainage performance, in addition to ensuring that its average porosity meets the technical requirements of the regulations [37], it is also necessary to ensure that the effective porosity of the pavement meets the drainage function requirements. In addition, considering that the permeability anisotropy inside the REV is mainly manifested in that the permeability along the vertical direction (*z* direction) is larger, while the permeability in the *xy* plane (*x* direction and *y* direction) is smaller, from the perspective of the bucket effect, in practical engineering applications, the drainage performance of the pavement can be improved by increasing the slope of the drainage pavement (such as cross slope or longitudinal slope). The KC equation is as follows:

(5) $$k=\frac{p^{3}}{c{\left(1-p\right)}^{2}S^{2}}$$

where *k* is the calculated permeability of porous media (μm^2^), *p* is the porosity of porous media (%), *c* is the Kozeny–Carman constant, and *S* is the specific surface area of the solid-phase pores.

### 4.3. Three-Dimensional Microscopic Morphological Characteristics of Connected Pores

Due to the complex morphology of the connected pores inside the PA mixture, water flows non-linearly in the throat, so it is difficult to analyze the void structure of all its seepage paths in detail. For this reason, the minimum section area, equivalent diameter and channel length of the throat, and tortuosity are usually used to characterize the 3D microscopic morphological characteristics of connected pores [44]. After calculation, the 3D void mesostructures parameters of the connected pores of REV-3 are shown in Table 5 and Table 6.

Minimum section area and tortuosity are significant factors for controlling water flow and drainage time, respectively. In general, the flow of liquid in the seepage channel with connected pores as carriers inside the porous asphalt mixture is similar to the flow of pipes with smaller diameters or the flow of capillary bundles. According to Darcy’s law formula *Q* = *K*·*A*·(Δ*h*)/*L* (where *Q* is the seepage amount per unit time, *K* is the permeability coefficient, *A* is the cross-sectional area in the direction of water flow, Δ*h* is the head difference, and *L* is the seepage path length), when the water head difference between the upper and lower bottom surfaces of the sample is constant, the seepage amount *Q* is directly proportional to the cross-sectional area *A* perpendicular to the direction of water flow and inversely proportional to the seepage length *L*. Therefore, the smaller the minimum section area of the narrow place of the seepage channel, the smaller the seepage amount per unit time *Q*, and the greater the tortuosity of the seepage channel, the longer the fluid seepage path *L*, resulting in a longer time for the fluid to flow through the upper and lower surfaces of the specimen under the same pore pressure and flow rate conditions. As listed in Table 5, the total number of throats in the equivalent pore network model is 3743; this is a large number, indicating that there are abundant seepage paths distributed inside the PA mixture. The maximum values of the ER and area of the throat are 85 times and 816 times their minimum values, respectively, which illustrates that the spatial morphological characteristics of the pores are relatively complex. The reason is that, due to the influence of many factors during the compaction and molding of the solid porous matrix, the morphological differences of the void mesostructures at different positions inside the mixture are very different. From Table 6, it was observed that the minimum section area of each vertical connected pore inside REV-3 generally ranges from 0.042 mm^2^ to 25.260 mm^2^, and the tortuosity is 1.42~1.66. The maximum values of the minimum section area and tortuosity are 601.43 times and 1.17 times their minimum values, respectively, indicating that the minimum section area of the connected pores of the porous asphalt mixture is very different, while the tortuosity parameters are relatively concentrated and the difference is small. Therefore, for the same kind of asphalt mixture, the influencing factor that causes significant differences in its permeability performance may be the difference in minimum section area. In order to improve the water permeability of the drainage pavement, during the material design and construction process of the drainage asphalt pavement, attention should be paid to increasing the cross-sectional area of effective pores inside the pavement structure while ensuring that the average void ratio or compaction of the pavement meets the specification requirements. For example, by adjusting the mineral aggregate gradation curve, reducing the amount of fine aggregate, increasing the roundness of coarse aggregate, limiting the flat-elongated particle content of coarse aggregate, and optimizing the mixing and compaction process, the connected void ratio or drainage performance of the porous asphalt pavement can be improved.

## 5. Conclusions

This study attempts to explore and characterize the three-dimensional mesoscopic void characteristics of porous asphalt mixtures. The 3D pore model of the PA mixture was reconstructed utilizing CT images and Avizo, the pore network model was extracted from REV, and the anisotropic distribution characteristics of connected pores were analyzed through corresponding evaluation indicators. The significant results obtained in this study can be summarized as follows:

- Based on MATLAB R2021b and Avizo 9.5.0 software, threshold selection and image processing were performed on the original CT scan image, image filtering was implemented, the optimal threshold for image segmentation was determined, and a three-dimensional numerical model of the specimen was constructed. The 3D model of the pore (REV-3) was extracted using mesomechanical analysis methods, model simplification, and the Axis Connectivity algorithm.
- The distribution pattern of cross-sectional porosity, the ER of the pore and throat, was quantitatively characterized through the anisotropic mesoscopic evaluation index. The results showed that the cross-sectional porosity is mainly distributed between 20% and 25%, and about 90% of the macropores have a diameter between 0.5 mm and 3 mm. The distribution of porosity is uneven along the REV height direction. As the smallest cross-section of the seepage path, the ER of the throat is mainly between 0.1 mm and 1.5 mm, which is much smaller than the ER of the pore.
- The topological spatial structure of pores is quite different, and their coordination numbers are mainly concentrated within 18. The pores with coordination numbers 1 to 10 constitute the main body of the pores inside REV, accounting for over 98% of the total number of pores. In addition, the permeability calculation results show that there is a significant difference in the permeability of each axis of REV compared to the total permeability of the specimen, which illustrates that the permeability distribution of REV presents an obvious spatial anisotropy. In order to further explore the anisotropic seepage mechanism of drainage asphalt pavement at the mesoscopic scale, in the follow-up work, a numerical simulation study of pore seepage will be carried out based on the three-dimensional reconstruction model of a real porous asphalt mixture specimen.
- The three-dimensional morphological characteristics of connected pores were analyzed based on parameters such as minimum section area, throat equivalent diameter, throat length, and tortuosity. The results exhibit that the minimum section area and tortuosity of connected pores have a greater impact on the seepage characteristics of porous asphalt mixtures. Compared with the latter, the minimum section area has a more significant impact on the water seepage performance of the PA mixture.

## Figures and Tables

**Table 1:** Basic physical parameters of the PA-16 specimen.

Property	Value
Bitumen-aggregate ratio (%)	4.7
Porosity (%)	24
Connected porosity (%)	21.8
Bulk volume density (g·cm^−3^)	2.01
Vertical osmotic coefficient (mL·(15 s)^−1^)	860

**Table 2:** Industrial CT scanning parameters of GE Vtomex.

Property	Value
Scanning voltage (kV)	200
Scanning mode	cross section
Imaging area (mm)	106 × 106
Resolving power (μm)	65
Maximum sample size (mm)	120 × 250 (*φ* × *h*)
Detection time (min)	60

**Table 3:** Comparison of effective porosity calculated with test results.

Threshold	Pore Volume (mm^3^)	Total Volume (mm^3^)	Calculated Effective Porosity (%)	Tested Effective Porosity (%)	Error (%)
*T*_1_ (68)	32,401	145,193	22.32	21.76	2.57
*T*_2_ (66)	31,977	145,193	22.02	21.76	1.19
*T*_3_ (54)	29,424	145,193	20.27	21.76	6.85
*T*_4_ (60)	30,714	145,193	21.15	21.76	2.80
*T*_5_ (61)	30,920	145,193	21.30	21.76	2.11
*T*_6_ (63)	31,358	145,193	21.60	21.76	0.74
*T*_7_ (64)	31,565	145,193	21.74	21.76	0.09
*T*_8_ (65)	31,771	145,193	21.88	21.76	0.55

**Table 4:** Statistics of pore parameters in the equivalent pore network model.

Total Pore Count	Pore ER (mm)			Coordination Number			Pore Area (mm^2^)		
	Max	Min	Ave	Max	Min	Ave	Max	Min	Ave
1977	4.24	0.14	1.36	18	1	3.79	368.59	0.30	43.21

**Table 5:** Statistics of throat parameters of the equivalent pore network model.

Total Pore Count	Throat ER (mm)			Throat Length (mm)			Throat Area (mm^2^)		
	Max	Min	Ave	Max	Min	Ave	Max	Min	Ave
3743	5.10	0.06	1.02	69.34	55.31	58.58	81.64	0.01	4.41

**Table 6:** Detailed characteristics of vertically connected pores in REV-3.

Minimum Section Area (mm^2^)	Equivalent Diameter (mm)	Channel Length (mm)	Tortuosity
0.042	0.23	60.34	1.51
0.586	0.86	66.49	1.66
1.219	1.25	61.52	1.54
2.136	1.65	56.80	1.42
4.502	2.39	58.65	1.47
6.325	2.84	59.92	1.50
10.763	3.70	63.12	1.58
13.701	4.18	56.67	1.42
19.923	5.04	59.60	1.49
25.260	5.67	58.03	1.45

## Data Availability

The data that support the findings will be available from the corresponding author upon reasonable request.
